# Catheter Ablation for Atrial Fibrillation

**Published:** 2003-10-01

**Authors:** Andre J Gauri, Bradley P Knight

**Affiliations:** Division of Cardiology, Department of Internal Medicine, University of Chicago, Chicago Illinois

**Keywords:** Atrial Fibrillation, Catheter Ablation

Atrial fibrillation (AF), the most common arrhythmia in adults, affects 1 in 25 people over the age of 60 years and 1 in 10 over the age of 80 years [1]. There is considerable morbidity, mortality and economic burden associated with AF, all of which will increase with the expanding elderly population. Until recently, pharmacologic therapy with AV nodal blocking agents, antiarrhythmics and anticoagulation were the mainstay of therapy. Although electrical cardioversion is associated with a high immediate success rate, most patients have recurrences of AF with only 23% remaining in sinus rhythm one year after cardioversion [2]. Antiarrhythmic agents have been shown to improve sinus maintenance, but these medications have variable success and are associated with many potentially serious side effects. In addition, the recently published AFFIRM trial suggests that a pharmacological rhythm control strategy has no benefit in terms of mortality or morbidity over a rate control and anticoagulation strategy [3]. Over the last few years, there has been a great deal of enthusiasm regarding catheter based ablation strategies aimed at curing AF.

## Percutaneous Maze Procedure

In 1959 Moe theorized that AF resulted from multiple wavelets of reentry. With this in mind, Cox performed the surgical Maze procedure in 1991 [4]. The Maze procedure alters the arrhythmogenic substrate by interrupting the macroreenterant circuits and reducing the critical mass of atria needed to sustain AF. This surgical approach currently is preformed in association with coronary bypass surgery and/or mitral valve repair and is successful in curing AF in 75-90% of cases [5]. Although the surgical procedure has been modified over time, the approach has consistently included isolation of the pulmonary veins (PV). Currently, malleable hand-held catheters are being used to create linear lesions with conventional radiofrequency energy, rather than surgical incisions.

**Figure 1:**
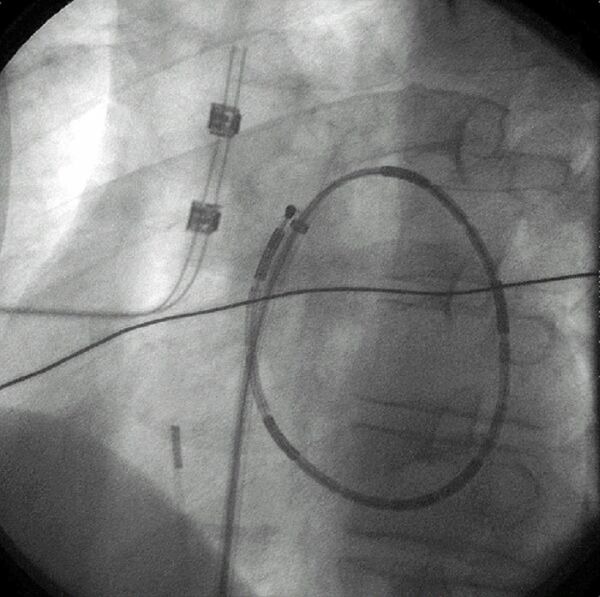
A specially-designed, multiple-electrode loop ablation catheter (EP Technologies) is shown deployed in the left atrium during an endocardial, catheter-based Maze procedure. The view is left anterior oblique. The catheter emerges from the transeptal sheath and a loop is created after the tip of the catheter is pulled to the end of the sheath with a pull-wire as the body of the catheter is advanced into the atrium. There are 14 individual ablation coils on the catheter. Coils number 1, 4, 7, 8, 11, and 14 are more radiopaque to improve identification. An intracardiac echo probe can be seen in the body of the right atrium.

**Figure 2:**
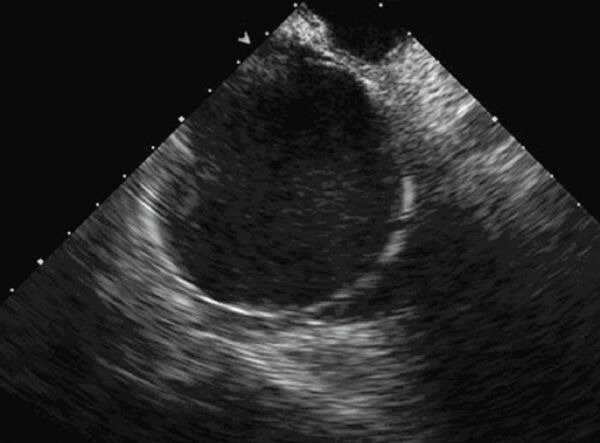
A view of the multiple-electrode loop ablation catheter deployed in the left atrium as seen from a phased-array intracardiac echo probe located in the right atrium. A subset of the individual electrode coils can be identified. Intracardiac echo can be useful during left atrial catheter ablation procedures to optimize tissue contact.

**Figure 3:**
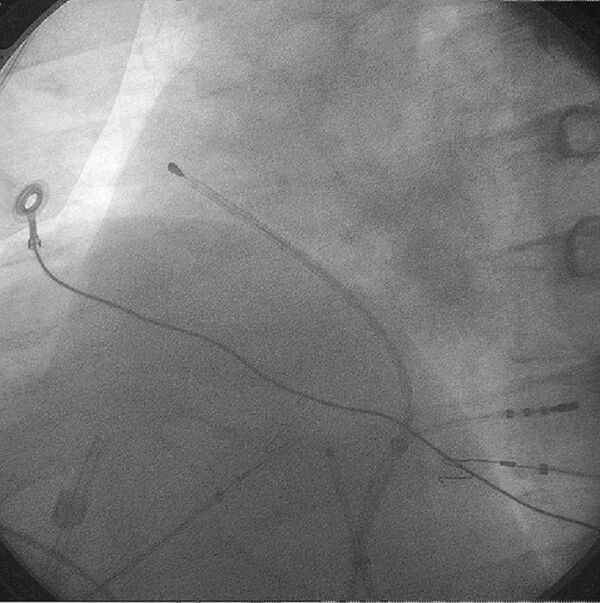
A 6-electrode deflectable ablation catheter (EP Technologies) is shown during an endocardial catheter-based Maze procedure. The view is left anterior oblique. The catheter courses along the right atrial septum from the coronary sinus to the anterior right atrium. A quadripolar electrode catheter is in the coronary sinus and an intracardiac echo probe is in the low right atrium.

The surgical experience over the past decade has provided evidence that the left atrium plays a significant role in the maintenance of AF and that a reduction in the left atrial mass prevents maintenance of AF. Because an open chest procedure is associated with significant morbidity, attempts have been made to replicate the Maze procedure using a percutaneous, catheter-based approach. The MECA (Multiple Electrode Catheter Ablation) trial, sponsored by Boston Scientific/EP technologies, was designed to determine the safety and feasibility of specially designed catheters with multiple large electrodes used to create circular, biatrial linear endocardial lesions to treat atrial fibrillation (Figure 1-3). The concept and catheter design were based on encouraging animal data [6]. However, the MECA study was terminated prematurely due to a relatively high complication rate and low efficacy rate [7]. Additional limitations of a percutaneous Maze procedure include a long, technically difficult procedure associated with long fluoroscopy times and the risk of proarrhythmia in the form of atrial tachycardias, which likely occur due to conduction gaps in the ablation lines. The role of linear atrial lesions in the treatment of atrial fibrillation remains unresolved.

## Importance of the Pulmonary Veins

Embryologically, the PVs form as a bud that grows from the heart towards the lungs. As a result, the PVs have a sleeve of muscle fibers that surround them. In the late 1990’s, Haissaguerre made a critical observation that the muscle fibers associated with the PVs are an important source of ectopic beats capable of triggering AF [8]. This discovery led to a revolution in interventional electrophysiology.

**Figure 4:**
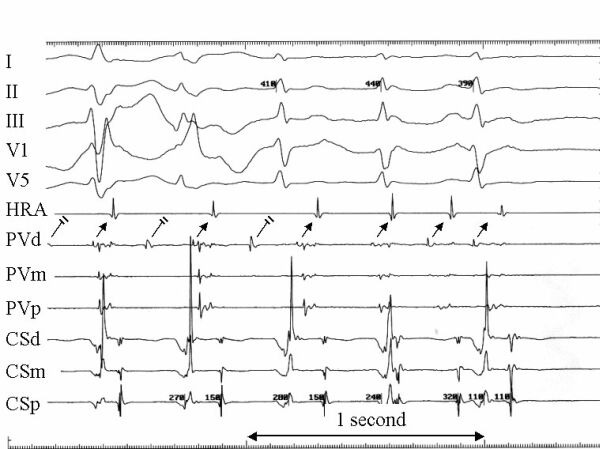
This tracing was recorded in a patient with an irregular, paroxysmal atrial tachycardia. Shown are surface recordings (I, II, III, V1, and V5) and intracardiac recordings from the high right atrium (HRA), the distal (PVd), mid (PVm), and proximal (PVp) electrode pairs on a catheter positioned at the ostium of the right superior pulmonary vein, and the distal (CSd), mid (CSm), and proximal (CSp) coronary sinus. The electrograms recorded at PVd show a rapid irregular pulmonary vein tachycardia from the right superior pulmonary vein with 2:1 conduction block to the left atrium. The conduction block resolves after a pause just prior to termination of the pulmonary vein tachycardia. Radiofrequency energy delivered at this site resulted in elimination of the tachycardia.

Pulmonary vein muscle tissue has unique electrophysiologic properties and appears to be able to maintain reentry within a relatively small amount of atrial muscle mass. This is likely due to the spatial complexity and a short refractory period. This area is also a common source of rapid focal discharges. Figure 4 shows an example of a rapid, irregular atrial tachycardia arising from a right upper PV that is associated with conduction block to the left atrium. The case highlights the unusual electrophysiologic observations that are made in the PVs. Although the arrhythmia is an atrial tachycardia in this case rather than AF, it is easy to see how a rapidly discharging focus in a PV could lead to AF by causing fibrillatory conduction or by initiating reentry in the atrium.

## Pulmonary Vein Isolation

**Figure 5:**
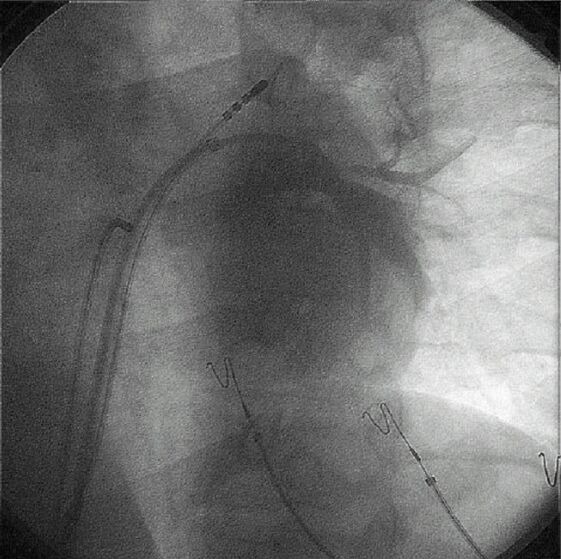
A pulmonary venogram is shown in a patient who had a prior focal ablation procedure for atrial fibrillation at which time radiofrequency current was delivered in the left inferior pulmonary vein. The view is left anterior oblique. The contrast injection shows a focal stenosis of a superior branch of the left inferior pulmonary vein. A quadripolar electrode catheter can be seen in the left superior pulmonary vein and along the right atrial septum. The patient was asymptomatic.

Initially, attempts were made to ablate the ectopic foci in patients who had paroxysmal AF that appeared to arise in the PVs. Despite high initial success rates, this approach soon proved to be inadequate with recurrence rates over 60% [9,10]. The limitations of a focal approach include the presence of multiple PV foci, a paucity of spontaneous or inducible AF during the procedure, the subsequent development of new foci, and the difficulty in mapping triggers when AF is persistent. In addition, delivery of radiofrequency energy deep within the PV can lead to PV stenosis ( Figure 5).

Electrical isolation of the pulmonary veins avoids many of the limitations of focal PV ablation. By isolating a PV from the atrium without ablating the entire circumference of the PV ostium, Haissaguerre and his group developed a technique referred to as ostial segmental PV isolation Figure 11. The myocardial sleeves that connect the PV to the left atrium are a complex network of fibers. These fibers do not completely encircle the venous os, but are typically located in segments or quadrants. Identification of these muscular sleeves allows selective radiofrequency (RF) energy delivery, thereby minimizing the risk of PV stenosis.

**Figure 6:**
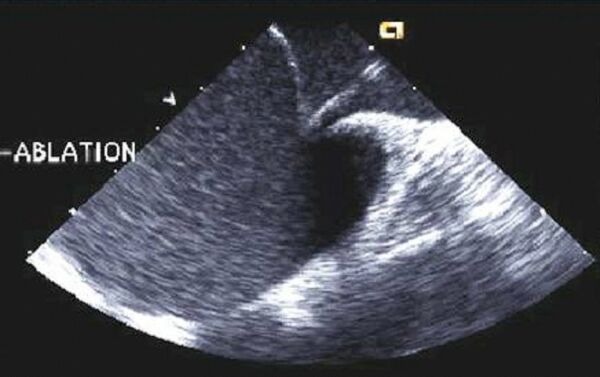
An intracardiac echo image during transeptal puncture. The transeptal needle can be seen “tenting” the thin part of the intratrial septum. Intracardiac echo can be useful to guide transeptal catheterization.

**Figure 7:**
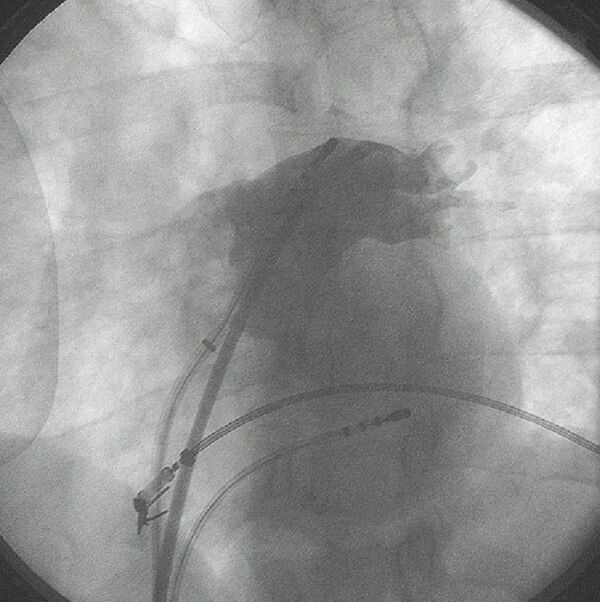
A pulmonary venogram is shown in a patient undergoing an ostial segmental pulmonary vein isolation procedure. The view is left anterior oblique. The contrast injection shows a common ostium of the left upper and lower pulmonary veins. There is a quadripolar electrode catheter positioned at the roof of the common ostium and one positioned in the coronary sinus.

The procedure requires transeptal catheterization. Intracardiac echocardiography can be useful to guide transeptal puncture ( Figure 6). Identification of the PV ostia using venography or intracardiac echo is important to identify anomalies, such as a common PV ostium ( Figure 7), and to avoid delivery of RF current in the PVs. Many centers perform a three-dimensional cardiac CT scan or MRI prior to ablation to screen for PV variants and to have a baseline imaging study in case a patient develops symptoms suggestive of PV stenosis after ablation.

**Figure 8:**
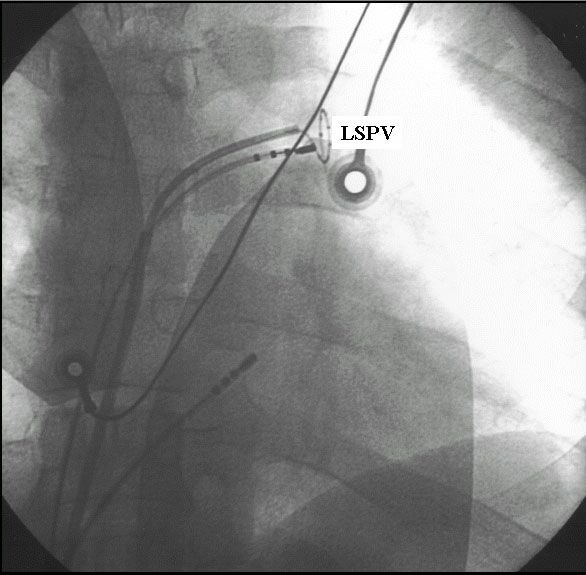
An anterior-posterior fluoroscopic view of a decapolar circular mapping catheter (Lasso, Biosense Webster Inc.) and a deflectable quadripolar 4-mm tip ablation catheter positioned at the ostium of the left upper pulmonary vein during a segmental ostial pulmonary vein isolation procedure. A third electrode catheter is positioned in the coronary sinus.

**Figure 9:**
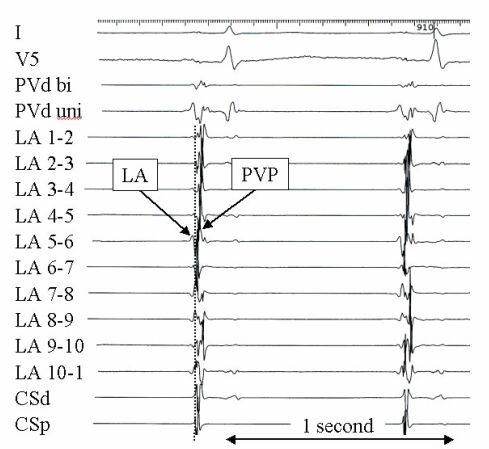
An example of pulmonary vein potentials (PVPs) recorded at the ostium of the right superior pulmonary vein during sinus rhythm prior to pulmonary vein isolation. Shown are surface leads I and V5 and bipolar intracardiac electrograms recorded by the decapolar circular mapping catheter (LA 1-2 to LA 10-1) and the distal (CSd) and proximal (CSp) coronary sinus. PVd bi and PVd uni represent the bipolar and unipolar electrograms recorded from the ablation catheter positioned at the ostium of the PV. The PVPs can be distinguished from the left atrial (LA) electrogram by their sharp appearance. There are several pulmonary vein potentials visible following the LA recording at the PV ostium. The earliest PVP appears to be electrode number six as highlighted by the vertical dotted line and was the first site targeted with ablation.

**Figure 10:**
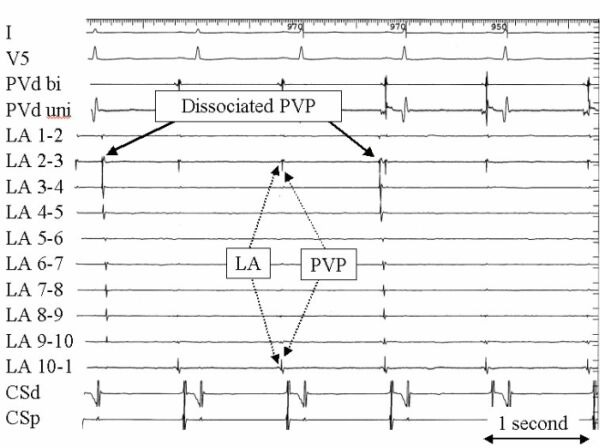
This tracing is an example of a pulmonary vein potential with spontaneous activity that is dissociated from the left atrium during sinus rhythm after partial ostial segmental pulmonary vein isolation. There remains an intact PVP recorded at electrodes 2-3 and 10-1. The format and abbreviations are the same as for figure 9.

**Figure 11:**
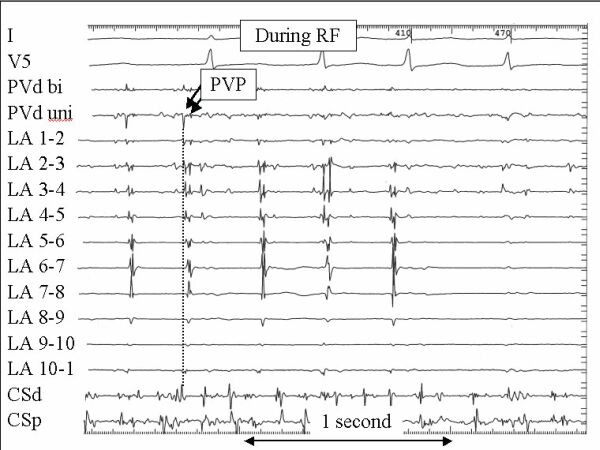
In this example radiofrequency current is delivered near electrode number 1 on the circular mapping catheter that is positioned at the ostium of the left superior pulmonary vein. There are two pulmonary vein potentials visible during atrial fibrillation with the earliest activation seen at LA 1-2 as highlighted by the vertical dotted line. All PVPs are eliminated during ablation. The format and abbreviations are the same as for figure 9.

**Figure 12:**
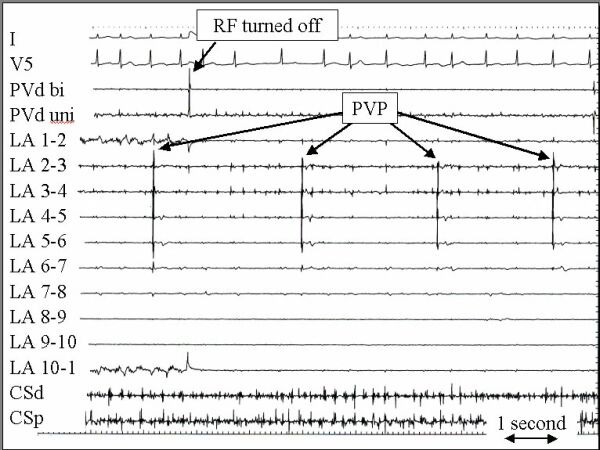
An example of a pulmonary vein potential with spontaneous activity that is dissociated from the left atrium during atrial fibrillation is shown after complete pulmonary vein isolation. The recording was made at the time that RF current was discontinued. The format and abbreviations are the same as for figure 9.

In order to identify PV potentials, a multiple electrode catheter is positioned near the ostium of the PV. This can be accomplished using a basket-type catheter or a circular multipolar mapping catheter. An example of a commonly used circular decapolar catheter is shown in figure 8. The decapolar catheter is positioned within 5 mm of the PV ostium, and RF energy is delivered at the ostial sites that display the earliest bipolar PV potentials during sinus rhythm or during coronary sinus pacing, or at sites that have the largest intrinsic deflections in unipolar recordings. Utilization of both unipolar and bipolar recordings during segmental ostial ablation is associated with increased efficacy and less RF energy delivery [12,13]. The endpoint of this procedure is elimination or dissociation of pulmonary venous potentials (Figure 9 -10). PV isolation is usually performed during sinus rhythm but can be accomplished during AF when sinus rhythm cannot be maintained initially during the procedure (Figure 11 -12). Initial strategies consisted of ablating only the arrhythmogenic PV’s, but empiric isolation of all four PV’s has been shown to be associated with a higher success rate.

Segmental ostial ablation has been associated with high success rates in patients with paroxysmal lone atrial fibrillation. Among the first 58 patients treated at the University of Michigan, 70% were free from AF recurrences and 83% were free of symptomatic AF or had significant improvement at 5 months follow-up [14]. Segmental ostial ablation appears to be less successful in patients with persistent or permanent AF. Only 22% of patients with persistent AF were free from recurrent AF at 5 months [14]. This high failure rate suggests that the left atrium plays a more important role in patients with persistent AF compared to patients with paroxysmal AF.

Recurrent AF after segmental ablation is likely due to recovery of conduction of the PV fibers. Other causes include ectopy arising from a small ostial cuff of muscle proximal to the ablation site, or non-PV ectopic foci arising from the superior vena cava, coronary sinus, left or right atrium, ligament of Marshall, or rarely the inferior vena cava [15]. As a result, 10-40% of patients undergoing segmental ostial ablation require a repeat ablation procedure [11,14].

In experienced centers, the risk of a major complication including PV stenosis, cardiac tamponade, stroke and death is approximately 2%. Measures that are used to minimize the risk of PV stenosis include delivery of energy only at the ostium, limitation of the power to 30-35 watts and temperature to 52° C, discontinuation of RF if no effect is seen within 20 seconds, periodic pulmonary venography, and intracardiac echo visualization and Doppler measurement. Although some degree of PV narrowing may occur following the ablation procedure, clinically relevant PV stenosis is rare. Three-dimensional computed tomography has been shown to be useful in the identification of pulmonary vein stenosis. A series from the University of Michigan found that among 58 consecutive patients who had undergone a segmental ostial isolation procedure, there were no patients with symptomatic PV stenosis [16]. Routine computed tomography scans performed four months following the ablation identified an average narrowing of 1.5 mm of the ostial diameter. A 28% to 61% stenosis was present at an average distance of 7.6 ± 2.2 mm from the ostium in only 3% of the 128 PVs treated. The Cleveland Clinic group recently reported the results of using routine spiral computed tomography to detect PV stenosis following PV isolation ablation procedures [17]. In their series of 335 patients, 18 (5%) had severe PV stenosis defined as a luminal narrowing of > 70%. However, only 10 of the patients (3%) were symptomatic with the most prevalent symptom being shortness of breath (8 patients), followed by cough (7 patients) and hemoptysis (5 patients). An awareness of this potential complication is important since therapeutic options are available. Balloon angioplasty and venous stenting have been performed in symptomatic patients.

Pulmonary vein isolation was reported initially to be associated with long procedure and fluoroscopy times. However, it has been shown that centers that have performed over 75 cases can typically complete the procedure in less than 3 hours and with less than 60 minutes of fluoroscopy [18].

## Electroanatomic Left Atrial Ablation

**Figure 13:**
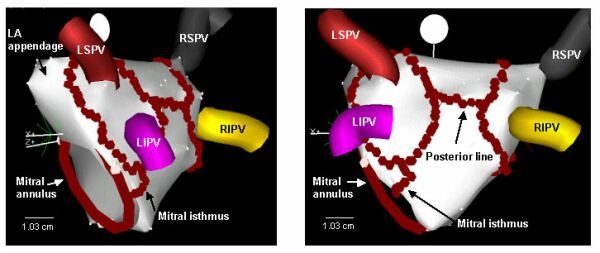
Electroanatomic maps of the left atrium during an electroanatomic left atrial ablation procedure for atrial fibrillation. The left superior (LSPV), left inferior (LIPV), right superior (RSPV), and right inferior (RIPV) pulmonary veins are labeled. The continuous series of solid red circles encircling the pulmonary veins represent radiofrequency ablation sites. Two additional linear lesion sets are shown: one connecting the two circular lesions that surround the pulmonary veins, and one connecting the mitral valve annulus to the circular lesion that surrounds the left pulmonary veins. (Figure courtesy of Dr. Fred Morady)

An alternative endocardial ablation technique to treat AF has been described by Pappone. This technique uses a circumferential electroanatomic approach [19]. The procedure described by Pappone involves a 3-D electroanatomic mapping system (CARTO, Biosense Webster Inc.) to map the atria and PVs. Circumferential RF lesions are then created at 5 mm from the PV ostia. This anatomic approach eliminates the need for mapping spontaneous or induced arrhythmias. The end point for ablation is a bipolar amplitude less than 0.1 mV inside the lesion and a delay of greater than 30ms across the ablation line (Figure 13). The one-year success rates, defined as freedom from AF, obtained by the Pappone group in 251 patients (paroxysmal AF=179, permanent AF=72) treated was 80% overall, with 86% for paroxysmal AF and 68% for permanent AF [20]. Only 75% of the circumferential lesions surrounding the individual PVs met criteria for complete, defined as a bipolar amplitude < 0.1 mV. Interestingly, they found no relation between lesion completeness and clinical outcome. This finding led the Pappone and his group to use the term “electroanatomic remodeling” to describe the alteration in the atria substrate that occurs during this ablation technique which prevents atrial fibrillation. This approach of PV isolation plus substrate modification may explain the higher success rates obtained compared to exclusively isolating the PV and eliminating the “trigger” of AF in the segmental ostial ablation approach. Which of these two approaches is superior is a source of debate. A randomized control trial comparing segmental ostial ablation to circumferential ablation in patients with paroxysmal AF is currently underway.

## Conclusions

The last few years have marked the beginning of an exciting new era in the treatment for AF. For patients with paroxysmal AF, both segmental and circumferential ablations appear to have comparable long term success rates and low rates of complications. However, for patients with persistent or permanent AF, the circumferential ablation approach using 3D-elcctroanatomic mapping appears to be more successful. Patients with AF who are suitable candidates for catheter ablation are those with symptomatic AF despite reasonable pharmacologic efforts and minimal structural heart disease. The future of ablation therapy for AF will likely be an approach which both eliminates the trigger of AF and alters the substrate which permits maintenance of the arrhythmia. New catheter designs and alternative energy sources are currently under investigation to improve the safety, efficiency, and success rate of catheter ablation for AF.
